# Supplementary material for: Polymorphisms in Pfkelch13 domains before and after the introduction of artemisinin-based combination therapy in Southwest Nigeria
Source: PLoS One. 2025 Mar 31;20(3):e0316479. doi: 10.1371/journal.pone.0316479 (PMC11957316; doi:10.1371/journal.pone.0316479)

Samples: 13093
Bases: 1114
Average spacing: 12

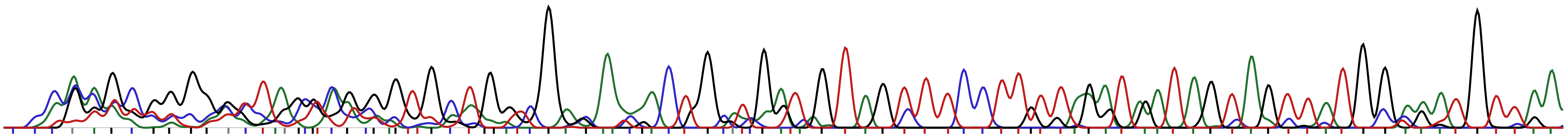

10 20 30 40 50 60 70 80 90

C C C - A G C G G G G - C T A - G C G T C G C G A G T T G C T G G A T C G A G A A C G A C T G C - T C G A T A G T A G T T T C C T T G T T A G A T A A T A G T A G T T A T G G A A A T G T T A A

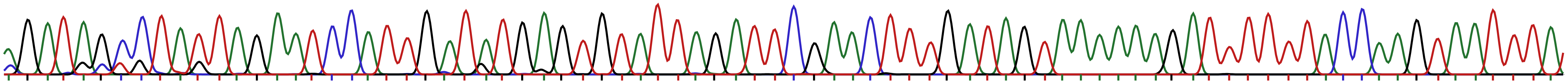

100 110 120 130 140 150 160 170

A G A T A G C C T A T T A G A A T C C A T T G A T A T G A G T G T A T T A G A T T C G A A C T T T G A T A G T A A A A A A G A T T T T T T A C C A A G T A A T T T A

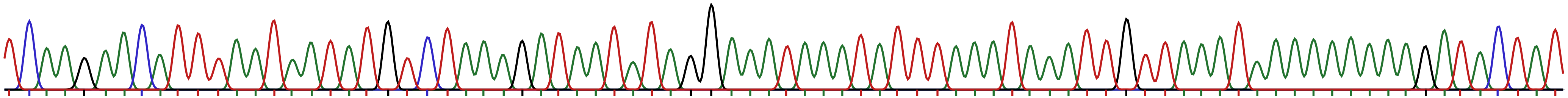

180 190 200 210 220 230 240 250 260

T C A A G A A C A T T T A A T A A T A T G T C T A A A G A T A A T A T A G G A A A T A A A T A T T T A A A T A A A T T G T T A A A T A A A A A A A A A G A T A C T A T

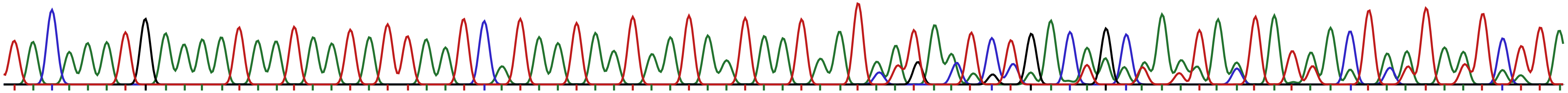

270 280 290 300 310 320 330 340

T A C A A A T G A A A A T A A T A A T A T T A A T C A T A A T A A T A A T A A T A A T A A T A A T A A T A A T C T G A C A G C A A A T A A T A T A A C T A A T A A T C T T A

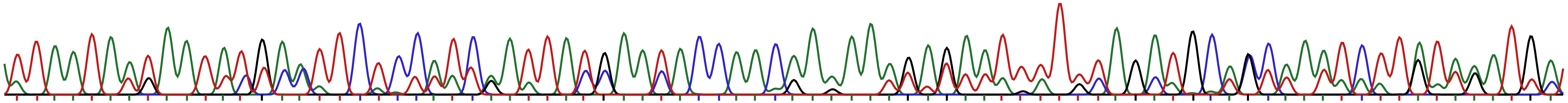

350 360 370 380 390 400 410 420

T T A A T A A T A A T A T G A A T T C T C C A T C A A T T A T G A A T A C C A A C A A A A A A G A G A A T T T T T T T A G A T G C A G C A A A T C T T A T A A A T G A

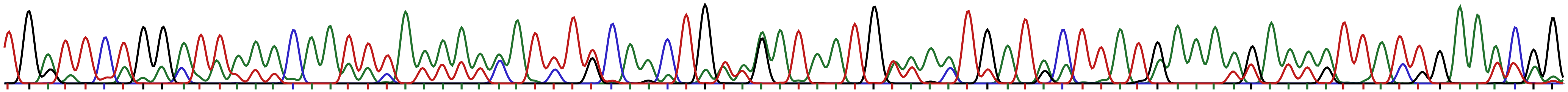

430 440 450 460 470 480 490 500

T G A T T C T G G A T T A A A C A A T T T A A A A A A A T T T T T C A A C T G T A A A T A A T G T A A A T G A T A C T T A T G A A A A G A A A A T T A T T G A A A C G G

Samples: 13093
Bases: 1114
Average spacing: 12

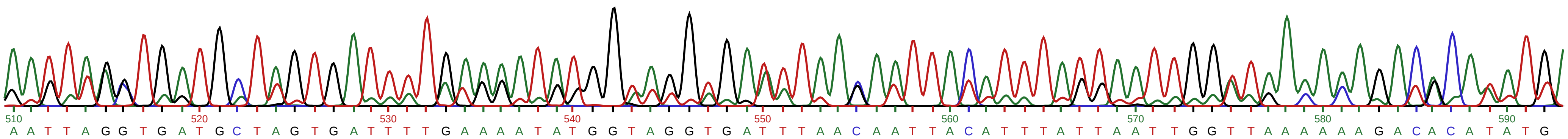

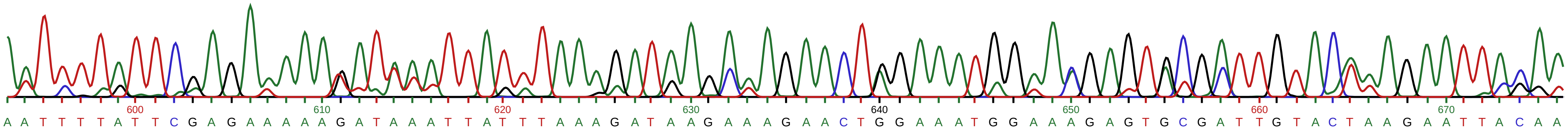

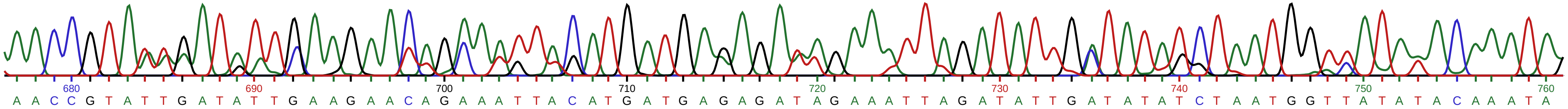

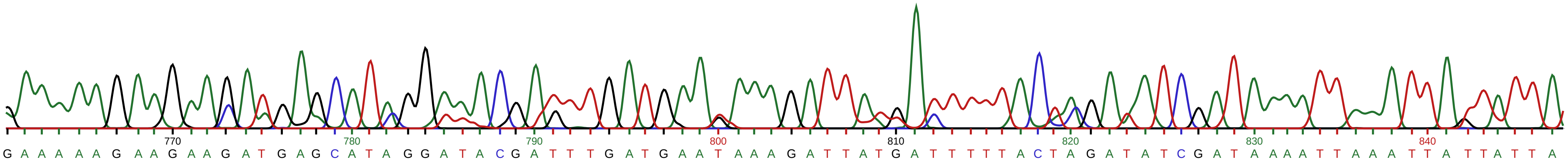

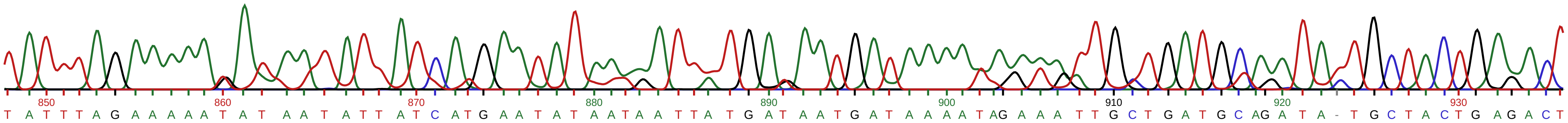

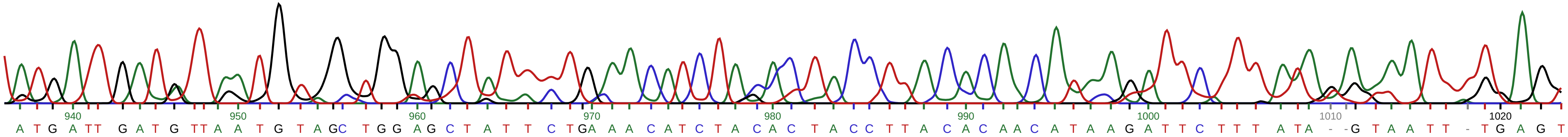

Samples: 13093
Bases: 1114
Average spacing: 12

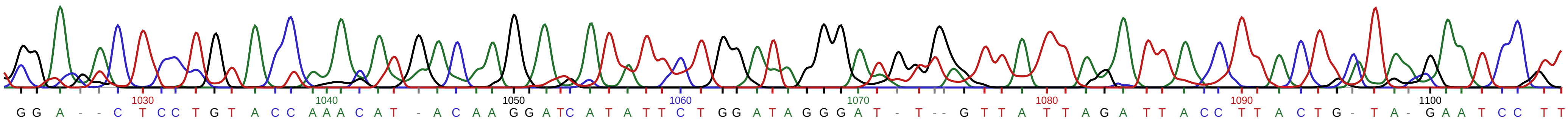

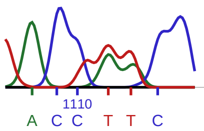

Supplement: Supporting information 2 — (ZIP) [file pone.0316479.s002.zip › 051KN2F_PREMIX_Plate_KELCH2_A06.pdf]
